# Prevalence and factors associated with depression among older adults in the case of a low-income country, Ethiopia: a systematic review and meta-analysis

**DOI:** 10.1186/s12888-022-04282-7

**Published:** 2022-11-01

**Authors:** Ayele Semachew Kasa, Shu-Chun Lee, Hui-Chen (Rita) Chang

**Affiliations:** 1 School of Nursing, Faculty of Science, Medicine and Health, University of Wollongong, PO Box: 53, Porter St. North Wollongong, NSW Australia; 2 Illawarra Health and Medical Research Institute (IHMRI), Wollongong, New South Wales Australia; 3 Department of Adult Health Nursing, School of Health Science, College of Medicine and Health Sciences, Bahir Dar University, Bahir Dar, Ethiopia; 4 School of Gerontology Health Management, Taipei Medical University, Taipei, Taiwan

**Keywords:** Depression, Prevalence, Predictors, Risk factors, Older adults, Elderly, Ethiopia

## Abstract

**Background:**

Depression is among the common mental health problems in late-life and an important public health problem. Studies from both middle- and high-income countries have shown that depression is more common among older people than in adolescents. Many older people with depression are overlooked, and fewer efforts are made to mitigate their suffering. Despite depression being a major public health problem among older adults, its overall magnitude, and its main predictors were not determined for the development of appropriate measures. Hence, the objective of this study was, therefore, to estimate the overall prevalence of depression and identify its predictors among older adults in Ethiopia.

**Methods:**

Available articles were searched by means of different databases using the PRISMA guideline. The quality of the included studies was assessed using a JBI quality appraisal tool. STATA version 14.0 (STATA Corporation, College Station, Texas, USA) statistical software was used to analyze the eligible studies. Subgroup and sensitivity analyses were performed. Cochran’s Q and the I^2^ test were used to assess heterogeneity. The presence of publication bias was evaluated by using Egger’s test and visual inspection of the symmetry in funnel plots.

**Result:**

In this meta-analysis, we included 11 articles that assessed 6521 older adults. The overall prevalence of depression among older adults in Ethiopia was 41.85 (33.52, 50.18). The finding was higher in the Oromia region with a prevalence of 48.07% (95% CI: 35.62, 60.51). The finding also demonstrated that being female (AOR = 1.76, 95% CI: 1.17, 2.63), no formal education (AOR = 1.82, 95% CI: 1.03, 3.19), with chronic diseases (AOR = 2.46, 95% CI: 1.00-6.06), and no social support (AOR = 2.01, 95% CI: 1.06, 3.83) were found to be independent predictors of depression in older Ethiopian adults.

**Conclusion:**

Our systematic review and meta-analysis showed that almost two out of five older adults had depression. Female sex, no formal education, having chronic diseases, and no social support were the independent predictors of depression among older adults in Ethiopia. The study emphasizes that depression among older adults in Ethiopia calls for appropriate screening and interventions to reduce the occurrence and its overwhelming consequences.

**Supplementary Information:**

The online version contains supplementary material available at 10.1186/s12888-022-04282-7.

## Background

Depression is among the common mental health problems in older adults and it is among the major public health problems [1]. When people age, they will develop various comorbidities. In addition, when people age, they often spend more time alone. Due to these and other factors, older adults are at an increased risk for depression [2–4]. Moreover, depression reduces an individual’s productivity and ability to engage in daily activities of life, and it can even lead to suicide [5]. Depression and suicide in the older adults, 60 years and above, is a major global public health concern [6]. Suicide in older adults is often attributed to the development of depression due to bereavement or loss of physical health and other comorbidities [7]. A literature from United States (US) revealed older people make up 12% of the US population, but account for 18% of all suicide deaths secondary to depression and other comorbidities [8].

Depression is among the common mental health problems characterized by low mood, loss of interest or pleasure, decreased energy, feelings of guilt or low self-worth, disturbed sleep or appetite, and poor concentration [9, 10]. Compared with younger people older adults with depression often manifests differently [11]. Older people may not exhibit obvious symptoms of depression. Instead, they may feel tired, have difficulties sleeping, feel irritable, feel confused, have difficulties concentrating, fail to enjoy activities that they used to, move slowly, and experience changes in weight or appetite, in addition to feeling hopeless, worthless, or guilty [12]. These symptoms are common in older people, and they are often overlooked during the early stage of depression in an older adult.

Depression is a leading cause of disability worldwide, and it is the fourth largest contributor to the overall global burden of disease [13]. It affects people of all genders, ages, and backgrounds in communities worldwide [14–16]. The 2021 Institute of Health Metrics and Evaluation report revealed that approximately 280 million people worldwide have depression [17]. Studies from middle and high-income countries [18, 19] have revealed that depression is more common among older people than adolescents. It is a common illness worldwide and affects an estimated 3.8% of the global population, of which 5.7% are older adults older than 60 years of age [20].

Research has identified multiple factors that are associated with depression in older adults [21]. Sociodemographic factors such as older age, female sex, widowhood, a separated/divorced marital status [22, 23], lack of formal education, living alone [24], and poor economic status have been highlighted by multiple studies [25–27]. In addition, low quality of life [21, 22, 25], psychological stress [28, 29], functional impairment [21, 25, 30], poor social support [23], chronic diseases [24, 29], and cognitive impairment [29, 31] have been identified as risk factors for depression among older people.

Even though there are effective ways to prevent and treat depression in later life, the changes of aging can lead to depression [32]. However, many older people with depression are overlooked, and less efforts made to mitigate their suffering [33]. If left untreated, depression in older adults may result in the onset of physical, cognitive, functional, and social impairment, as well as decreased quality of life, delayed recovery from illness, increased health care utilization, and suicide [34]. Hence, determining the prevalence and identifying associated factors of depression among older adults at a country level will help in alerting healthcare providers to give emphasis and concern in screening and finding ways to lessen depression in older adults in Ethiopia.

Regardless of the age of the study population, depression is regarded as the third most common disease burden in Ethiopia [35]. This is the highest proportion of burden compared with other forms of mental disorders [26]. Previous studies reported an epidemiologic variation of depression among older adults between 26.7% [36] and 68.10% [31] and that has varied over time and across geographical regions.

The exiting studies didn’t determine the overall magnitude of depression and its main predictors at a country level for the development of appropriate measures. Thus, this systematic review and meta-analysis is aimed to estimate the overall magnitude of depression and its associated factors among older adults in Ethiopia. Our study addressed the following two research questions. (1) What is the pooled prevalence of depression in Ethiopia? (2) What are the factors of depression in the older adults living in Ethiopia?

## Methods

### Reporting

The Preferred Reporting Items of Systematic Reviews and Meta-Analysis (PRISMA) checklist was used to report the results of the present systematic review (Additional file 1). In addition, the PRISMA flow chart was utilized to show the selection process for the studies included in the present analysis [37].

### Searching strategies

A search strategy was developed under the guidance of an experienced university librarian. Studies and gray literature in the subject area that reported on outcomes of interest were identified. The PubMed, Web of Science, SCOPUS, CINAHL, PsycInfo, WHO Global Index Medicus, and Hinari databases were searched to identify relevant studies published between these databases’ inception and October 20, 2021. In addition, the research repositories of Addis Ababa University and Bahir Dar University were searched using the search terms “prevalence,” “magnitude,” “epidemiology,” “factors,” “predictors,” “depression,” “elderly,” “old age,” and “Ethiopia.” Search strings were established using “AND” and “OR” Boolean operators. We also checked the reference lists of the retrieved studies for relevant studies that could be included in the current review. In addition, a manual search was conducted using the names of each region of Ethiopia.

## Eligibility criteria

### Types of studies

Original research studies that reported the prevalence and or predictors of depression in older adults in Ethiopia were included. Thesis reports or dissertations that reported outcome variables were not available during our search. Studies that were published as review articles, conference abstracts, editorials, commentaries, and articles without full text were not considered.

### Types of participants, outcomes, and context

In the present systematic review, the included study participants were older adults, defined as individuals aged 60 years or older [38, 39]. In this review, studies were included if they determined the prevalence of depression or identified the predictors of depression among older adults living in Ethiopia in their studies. The review considered studies that were conducted in community settings, outpatient clinics of hospitals, and residential aged care facilities.

### Assessment of methodological quality

Relevant studies were also evaluated for methodological quality before they were included. The two reviewers (ASK and HCC) independently assessed the risk of bias in each study by using the Hoy 2012 tool [40], which was designed to assess the quality of prevalence studies. The Joanna Briggs Institute (JBI) critical appraisal checklist, which is a standardized critical appraisal instrument for assessing prevalence studies [41], was utilized to appraise the methodological quality of the selected studies. The checklist comprises nine items. A score was assigned for each item (0 for a “not reported” or “not appropriate” response and 1 for a “yes” response); the scores of the items were summed to obtain a total quality score of between 0 and 9. Studies were then classified as being of low, moderate, and high quality when the obtained score was between 0 and 4, between 5 and 7, and either 8 or 9, respectively [42]. Studies that had high or moderate quality were included in the final analysis (Table 1). Any disagreements between the reviewers were resolved through discussions or consultations with the third reviewer (SCL) until a consensus was reached.

**Table 1:** Critical appraisal of the included studies, 2021

Included articles		Criterion No (items included to appraise prevalence studies).									Total (%)	Overall quality
Author	Year	**1**	**2**	**3**	**4**	**5**	**6**	**7**	**8**	**9**	**%**	
Yimer YM. et al. (34)	2021	**√**	**√**	**√**	**√**	**√**	**√**	**√**	**√**	**√**	100	High
Mulat N. et al. (33)	2021	**√**	**√**	**√**	**√**	**√**	**√**	**√**	**√**	**√**	100	High
Jemal K. et al. (20)	2021	**√**	**√**	**√**	**√**	**√**	**√**	**√**	**√**	**√**	100	High
Abate T. et al. (51)	2020	**√**	**√**	**√**	**√**	**√**	**√**	**√**	**√**	**√**	100	High
Mezemir Y. et al. (49)	2020	**√**	**√**	**√**	**√**	**√**	**√**	**√**	**√**	**√**	100	High
Amha H. et al. (19)	2020	**√**	**√**	**√**	**√**	**√**	**√**	**√**	**√**	**√**	100	High
Bekele GT. et al. (50)	2020	**√**	**√**	**√**	**√**	**√**	**√**	**√**	**√**	**√**	100	High
Abdu AO et al. (52)	2020	**√**	**√**	**√**	**√**	**√**	**√**	**√**	**√**	**√**	100	High
Habte E. & Takele T. (27)	2018	X	**√**	X	**√**	X	**√**	**√**	**√**	**√**	66.7%	Medium
Mirkena Y. et al. (12)	2018	**√**	**√**	**√**	**√**	**√**	**√**	**√**	**√**	**√**	100	High
Girma M. et al. (48)	2016	**√**	**√**	**√**	**√**	**√**	**√**	**√**	**√**	**√**	100	High
√ =Yes, criterion fulfilled, X = No, criterion not fulfilled												

Item 1: Was the sample frame appropriate to address the target population? Item 2: Were study participants sampled appropriately? Item 3: Was the sample size adequate? Item 4: Were the study subjects and the setting described in detail? Item 5: Was the data analysis conducted with sufficient coverage of the identified sample? Item 6: Were valid methods used for the identification of the condition? Item 7: Was the condition measured in a standard, reliable way for all participants? Item 8: Was there an appropriate statistical analysis? Item 9: Was the response rate adequate?

### Data extraction

Data were extracted from the included studies by two independent reviewers (ASK and HCC). A standard data extraction form was designed and pilot tested before it was applied to each included study [43]. The extracted data included data on the setting, study design, sampling methods, number of study participants, age range, mean age, outcome measurements, and outcome status of each study.

### Data analysis

A statistical meta-analysis was conducted to analyze the collected data and determine the pooled prevalence of depression among older adults in Ethiopia. The data were analyzed using STATA version 14.0 (STATA Corporation, College Station, Texas, USA) software. Heterogeneity across the studies was assessed using the *I*^2^ test and Cochran’s *Q* test [44]. The thresholds of 25%, 50%, and 75% were used to indicate low, moderate, and severe heterogeneity, respectively [45, 46].

The included studies were summarized and presented using a table and a forest plot. Using *I*^2^ test and Cochrane *Q* statistics [47], we evaluated the presence of potential heterogeneity among the included studies.

The collected data were pooled, and the meta-analysis results provided a summary of depression statistics with 95% confidence intervals (CIs). Consequently, the individual study proportions were listed with their 95% CI values. Because we observed heterogeneity across the studies, a subgroup analysis was performed to assess the contribution of each study to overall heterogeneity. The presence of publication bias was evaluated by performing Egger’s test and a visual inspection of the symmetry in funnel plots [44, 48, 49].

## Results

### Search results

**Fig. 1:**
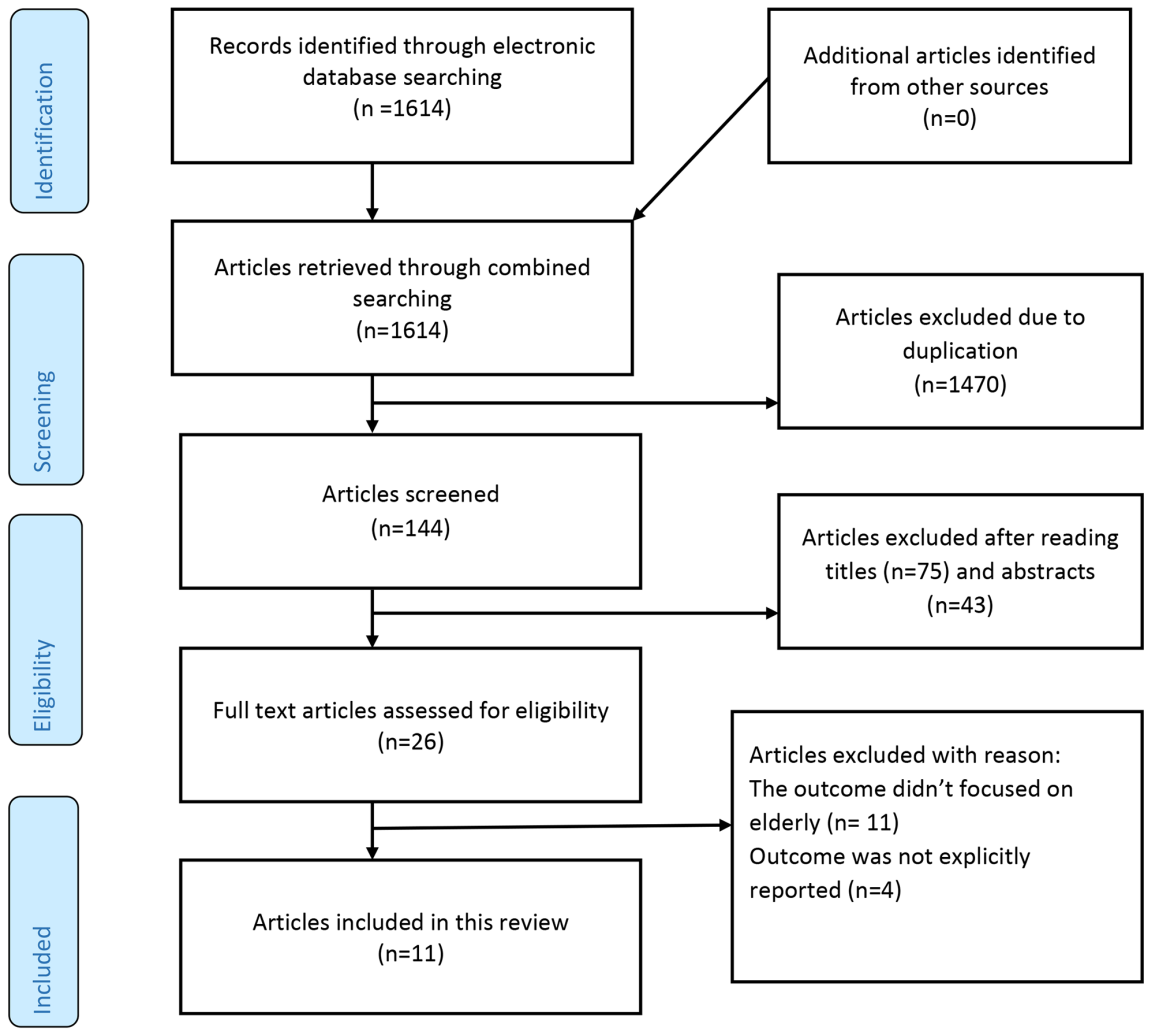
PRISMA Flow diagram on the prevalence of depression among older adults in Ethiopia, 2021

In this review, 1615 studies were retrieved by performing multiple forms of electronic searches on multiple databases. Among these studies, 1470 were excluded because they were duplicates, 75 studies were excluded after their titles were reviewed, 43 studies were excluded after their titles and abstracts were reviewed, and 16 studies were excluded for other reasons. Finally, 11 studies were included in this systematic review and meta-analysis. The inclusion process was conducted in accordance with the PRISMA flowchart (Fig. 1).

### Characteristics of included studies

In this study, 11 full-text studies were included. The included studies all utilized a cross-sectional study design. The number of study participants in each study ranged from 116 in a study conducted in Addis Ababa [31] to 941 in a study conducted in Amhara region [33]; the included studies had a total of 6521 study participants. The included studies were published between 2016 [50] and 2021 [24, 33, 36]. Four regional states (counties) of Ethiopia were represented in the studies. Four studies were conducted in the Amhara region [23, 33, 51, 52], three studies conducted in Addis Ababa [31, 36, 53], two studies were conducted in Oromia [1, 24] and two studies were conducted in Harar [50, 54]. Three studies [31, 53, 54] did not report the mean age of their study participants, whereas the mean age of the participants in the other included studies ranged from 66.69 to 75.46 years. The response rate of the included studies ranged from 93.4% [54] to 100% [24, 31]. All the included studies utilized the Geriatric Depression Scale-15 (GDS-15) as their outcome diagnostic measurement tool. Two studies recruited their participants from a residential aged care facility [31] and the outpatient clinic of a referral hospital [36], respectively, whereas the rest of the studies were conducted in community settings (Table 2).

**Table 2:** Characteristics of the included studies, 2021

Authors name	Publication year	Region	Study setting	Study design	Sampling method	% Of female	Age range	Mean age	No. of study participants	Response rate (%)	Outcome measures	Tot. No of outcome	Prevalence (%)	Do risk factors reported
Yimer YM. et al. (34)	2021	Addis Ababa	Inst.	Cross-sectional	SRS	36.4	60–80+	72.63	423	97.3	GDS-15	113	26.7	Yes
Mulat N. et al. (33)	2021	Amhara	Comnt (U & R)	Cross-sectional	MSS	50.8	60–75+	69.04	941	98.1	GDS-15	423	45	Yes
Jemal K. et al. (20)	2021	Oromia	Comnt (U & R)	Cross-sectional	MSS	48.7	60–90+	75.46	882	100	GDS-15	481	54.5	Yes
Abate T. et al. (51)	2020	Addis Ababa	Comnt.	Cross-sectional	SRS	63.7	65–85+	NR	662	97	GDS-15	183	27.64	No
Mezemir Y. et al. (49)	2020	Amhara	Comnt.	Cross-sectional	SRS	37.54	60–75+	69	341	98.27	GDS-15	214	62.8	No
Amha H. et al. (19)	2020	Amhara	Comnt (U & R)	Cross-sectional	SRS	59.3	60–75+	68.67	813	98.78	GDS-15	373	45.9	Yes
Bekele GT. et al. (50)	2020	Amhara	Comnt.	Cross sectional	SRS	57.8	60–80+	70.51	607	95	GDS-15	180	29.7	No
Abdu AO et al. (52)	2020	Harar	Comnt (U & R)	Cross-sectional	MSS	50.8	65+	NR	592	93.4	GDS-15	187	31.6	No
Habte E. & Takele T. (27)	2018	Addis Ababa	Inst.	Cross-sectional	Purposive	41.40	60–85+	NR	116	100	GDS-15	79	68.1	No
Mirkena Y. et al. (12)	2018	Oromia	Comnt.	Cross-sectional	MSS	45	60–75+	66.69	800	94.8	GDS-15	334	41.8	Yes
Girma M. et al. (48)	2016	Harar	Comnt.	Cross-sectional	SRS	61.9	60–75+	69.56	344	97.7	GDS-15	98	28.5	Yes

Comnt (U & R) = Community based study done both urban & rural, Comnt.= Community based study done in town, Inst.= Institution care centers, MSS = Multi-stage sampling, SRS = Systematic random sampling

### Prevalence of depression among older adults in Ethiopia

**Fig. 2:**
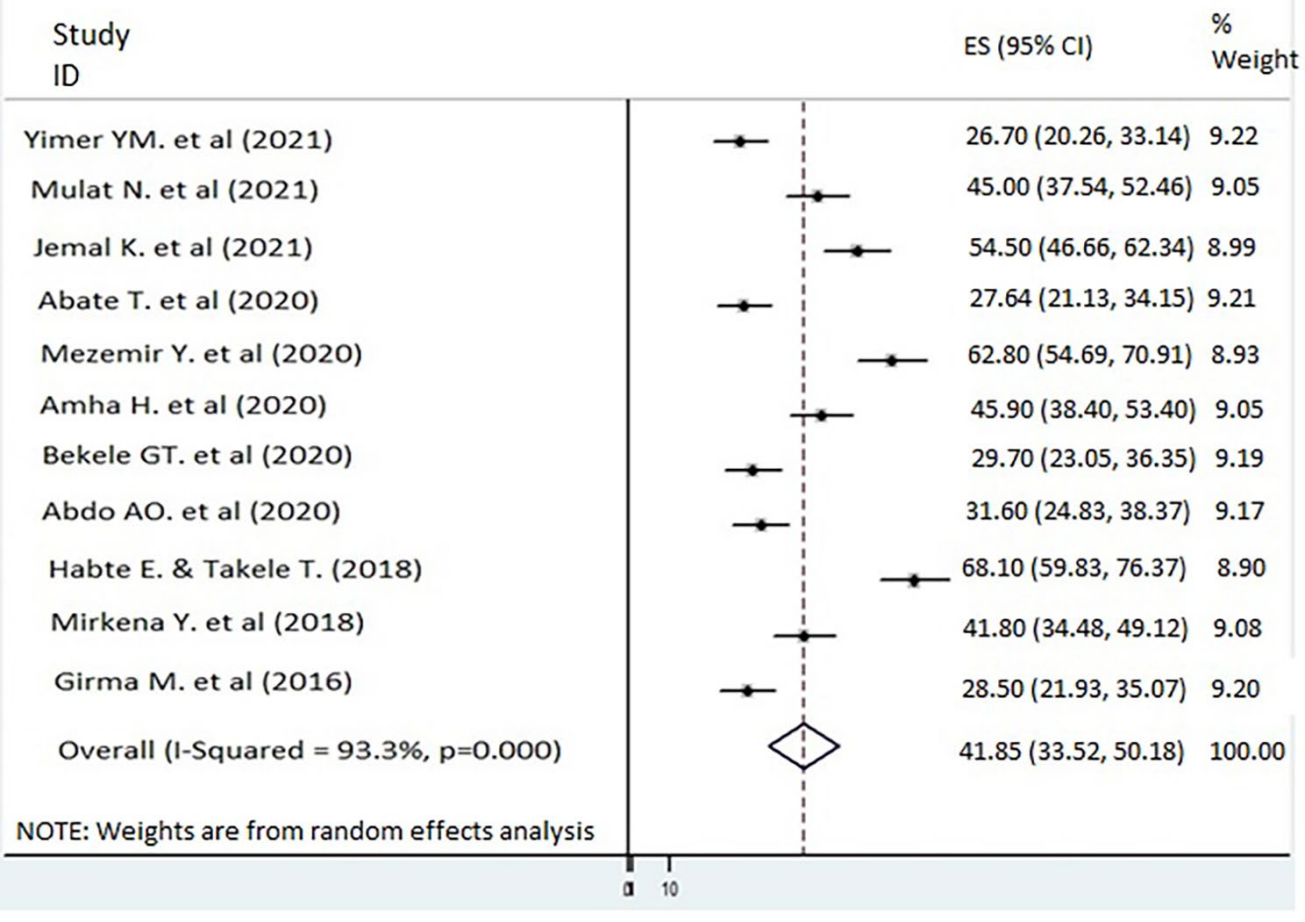
Forest plot showing the prevalence of depression among older adults in Ethiopia, 2021

Eleven studies were incorporated to estimate the overall prevalence of depression among older adults in Ethiopia. In the included studies, the minimum prevalence and maximum prevalence of depression among older adults were 26.7 [36] and 68.1 [31], respectively. The overall prevalence of depression among older adults in Ethiopia was 41.85 (95% CI = 33.52, 50.18; *I*^2^ = 93.3%; P < 0.001; Fig. 2). Because the test statistics revealed a considerable heterogeneity [55] across the studies (*I*^2^ = 93.3%, P < 0.001), a random effects model was used to estimate the Der Simonian and Laird’s pooled effect. To minimize the random variations between the point estimates of the included studies, a subgroup analysis was performed.

### Subgroup analysis

**Fig. 3:**
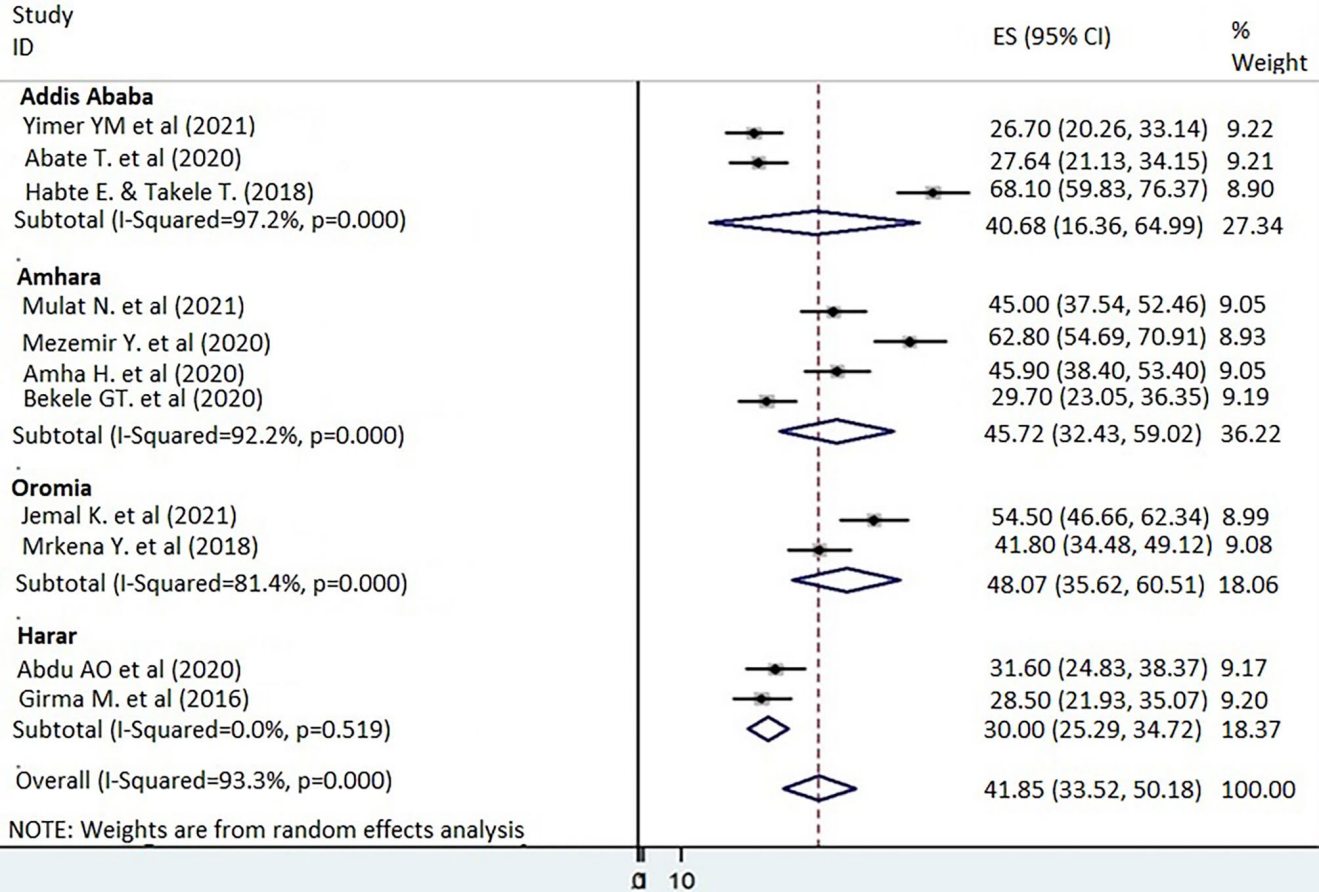
Subgroup analysis on the prevalence of depression among older adults by regions of Ethiopia, 2021

Because of a high level of heterogeneity across the included studies, a subgroup analysis was performed by region (county) and study settings in relation to outcome variables. The analysis revealed that the prevalence of depression among older adults was highest in the Oromia region (48.07%; 95% CI = 35.62, 60.51) followed by the Amhara region (45.72%; 95% CI = 32.43, 59.02) and Addis Ababa (40.68%; 95% CI = 16.36, 64.99), respectively (Fig. 3). Older adults who were recruited from institutions exhibited a higher rate of depression with a prevalence of 47.32% (95% CI = 6.74, 87.89), whereas older adults in community-based settings exhibited a lower rate of depression with a prevalence rate of 37.92% (95% CI = 25.9, 49.93; Additional file 2).

### Meta-regression

In addition to conducting the subgroup analysis, we also conducted meta-regression to identify the source of heterogeneity. A meta-regression analysis was conducted using the study variables of year of publication, number of study participants, and prevalence. However, its results revealed that none of the aforementioned variables were significant sources of heterogeneity.

### Publication bias

**Fig. 4:**
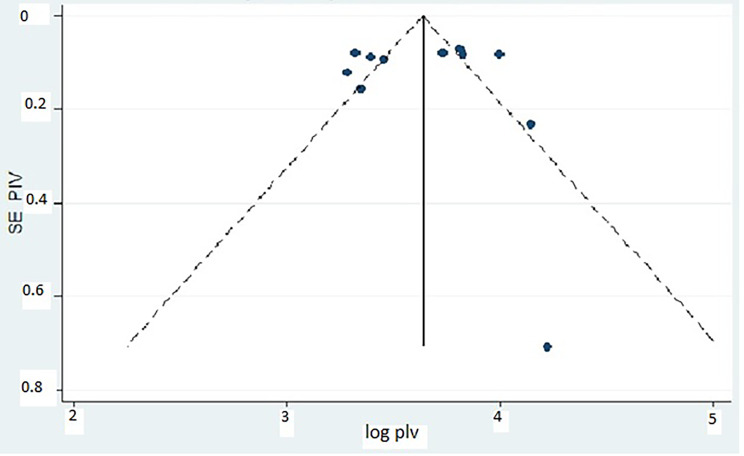
Funnel plot of the included studies on prevalence of depression among older adults, Ethiopia, 2021

Two methods for detecting publication bias in systematic reviews and meta-analysis were used. The funnel plot was visually inspected for publication bias (Fig. 4). In addition, publication bias was also objectively examined using Egger’s weighted correlation and Begg’s regression intercept test at a 5% significance level [44, 48, 49, 56, 57]. The test results indicated the absence of significant publication bias (β = − 0.22; standard error = 2.37; P = 0.93; Additional file 3).

### Factors associated with depression among older adults

Seven of the included primary studies on older adults reported factors associated with depression. From those studies, we extracted multiple factors. Among the extracted factors, having a chronic disease, having a lack of formal education, being female, being retired, being widowed, being divorced, and no social support were associated with depression among older adults. Four studies [1, 23, 33, 50] reported that female older adults were more depressed than male older adults. Three studies [23, 24, 50] reported that older adults with chronic diseases were more depressed than those without such diseases. Four studies [23, 24, 33, 36] reported that older adults with no social support were more depressed than those who received greater levels of social support. Two studies [24, 50] reported that older adults who did not receive formal education were more depressed than those who did.

In this study, being female (AOR = 1.76; 95% CI = 1.17, 2.63; Additional file 4), having a lack of formal education (AOR = 1.82; 95% CI = 1.03, 3.19; Additional file 5), having chronic diseases (AOR = 2.46, 95% CI = 1.00, 6.06; Additional file 6), and having no social support (AOR = 2.01; 95% CI = 1.06, 3.83; Additional file 7) were revealed to be independent predictors of depression among older adults in Ethiopia.

## Discussion

This study is the first systematic review and meta-analysis to estimate the pooled prevalence of depression and its associated risk factors among older adults in Ethiopia. In this systematic review and meta-analysis, 6521 older adults were included. The World Health Organization estimated that the overall prevalence rate of depressive disorders among the older adults in a given region or country generally varies between 10% and 20%, depending on cultural factors [58]. However, our study revealed that the pooled estimated prevalence of depression among older adults in Ethiopia was 41.85 (95% CI = 33.52, 50.18; *I*^2^ = 93.3%; P < 0.001). This finding is consistent with that of a study conducted in a neighboring country, Sudan, which reported that 41.1% of older adults had depression [59]. The results of our study are also relatively consistent with those of studies conducted in other African countries. Studies conducted in Ghana, Egypt, and Tanzania have revealed that 37.8% [60], 44.4% [61], and 44.4% [62], respectively, of older adults had depression. Furthermore, our study results are relatively consistent with those of a study that was conducted in Iran and that reported a depression prevalence of 43% among older adults [63].

Compared with our findings, those of other Africa-based studies have indicated a higher prevalence of depression among older adults. Studies conducted in Nigeria have indicated that between 45.5% [64] and 52% [65] of older adults have depression. The older adults examined in these Nigeria-based studies exhibited high functional disability [62], and more than one fifth of them were living alone [63]; these are factors that can increase the likelihood of depression in older adults. A study from South Africa also revealed that 51.9% of older adults had depression [66]. These older adults in South Africa were living with a human immunodeficiency virus infection or acquired immunodeficiency syndrome, which might have increased their likelihood of developing depression. Another study conducted in Egypt indicated that 62.7% of older adults had depression [67].

In contrast to the higher prevalence of depression observed in the aforementioned African countries, lower rates of depression were observed among older adults in other African countries. A study that examined populations in sub-Saharan countries revealed that 9.2% [67] of older adults had depression. Compared with our study, this sub-Saharan study included older adults who were aged 50 years or older and recruited them from multiple countries; these older adults varied in their socioeconomic characteristics and lifestyle. Studies conducted in Tanzania and Kenya have also revealed that 16.2% [68] and 22.9% [69], respectively, of older adults had depression. In addition to the study setting–related and age-related differences between these studies and the present review, differences in methodology were also noted. A South Africa–based study also reported that only 4% of older adults had depression [70]. This study had apparently recruited healthy community-based older adults who were aged 50 years or older. All the aforementioned factors might have contributed to the difference in the prevalence of depression among the participants of the South Africa–based study and that reported in our study.

Studies from Europe and Asia have also reported a high prevalence of depression among older adults. A study conducted in Vietnam reported that 66.9% of older adults had depression [71]. A study of older ethnic Turkish adults in the Netherlands revealed that 61.5% of them had depression [72]. In addition to variations in the tools used to measure outcome variables, these older adults in the Netherlands were migrants from Turkey. These migrant older people might have experienced diverse biopsychosocial constraints that caused their depression. A study from Palestine revealed that 51.9% of its study participants had depression [73]; in contrast to our study, it recruited older adults from refugee camps, which was a factor that could have contributed to the higher prevalence of depression among the older adults who were examined in that study. A study conducted in Nepal reported that 57.8% of older adults had depression [74]. This Nepal study recruited older adults who were not cared for by their children, which is a factor that could have increased the incidence of depression among these older adults. By contrast, most of the older adults that were included in our study were recruited from communities and had social connections.

However, other studies from various countries in Asia and Europe have revealed a low prevalence of depression among older adults. A study conducted in Europe revealed that 12.3% of older adults had depression [75]. A Malaysia-based [76] study reported that 7.6% of older adults had depression. A study from Japan [77] reported that 20.5% of older adults had depression. In addition to cultural and lifestyle differences, those studies utilized various diagnostic instruments to measure outcome variables. In the aforementioned studies, which were conducted in high-income and middle-income countries, the older adult participants were recruited from apparently healthy people who lived in community settings. In contrast to our study, a systematic review and meta-analysis conducted in Iran reported that only 8.2% of older adults had depression. This considerable difference between the finding of our study and that of the Iran-based study could be attributed to age. The Iran-based study included older adults aged 50 years or older, whereas our study included older adults aged 60 years or older. Furthermore, the Iran-based study only reported on older adults with severe depression. A Beijing-based study reported that 13% of older adults suffered from depression [75]. All of these reasons could have contributed to the lower levels of depression among the older adults reported by that study relative to those reported in our study [78].

Depression is the result of a complex interaction of social, psychological, and biological factors [13]. Our study revealed that being female is a risk factor for depression among older adults. This finding is consistent with those reported by studies conducted in Kenya [69], Nigeria [65], Ghana [79], Egypt [80], Iran [63], and the Netherlands [72], all of which also reported a significant association between female sex and depression among older adults. Our finding also revealed that older adults with no formal education were more likely to have depression. A study from Palestine also indicated that low educational status [73] was a predictor for depression among older adults. This systematic review and meta-analysis revealed that older adults with chronic disease were more likely to have depression. This finding is also consistent with those of studies conducted in South Africa [70], China, [78] and the Netherlands [72], all of which reported that older adults living with chronic medical conditions were more likely to have depression. In our study, we found that older adults with no social support were more likely to have depression. Similarly, studies from Japan [77] and Tanzania [62] have also indicated that older adults with favorable social support were less likely to experience depression.

A further challenge for research and clinical practices related to the diagnosis of depression in older adults involves the diagnosis of depression in individuals with cognitive impairment. Because the prevalence of cognitive impairment increases with age, the differential diagnosis of depression and dementia becomes increasingly difficult with age. Our review identified only two studies [50, 52] that assessed cognitive impairment among older people. The underlying neuropathological conditions that lead to mild cognitive impairment (MCI) or dementia may also contribute to depression; thus later-life depression, MCI, and dementia may fall in a clinical continuum [81].

## Conclusion and recommendation

Our systematic review and meta-analysis revealed that depression among older adults in Ethiopia is now a public health problem, and appropriate screening and interventions should be implemented to reduce its occurrence and considerable effects on older people in Ethiopia. Our findings also revealed that being female, having a lack of formal education, having chronic diseases, and absence of social support were independent predictors of depression among older adults in Ethiopia.

The WHO developed brief psychological intervention manuals for depression that lay workers can apply to individuals and groups [13]. Therefore, healthcare facilities and health care professionals in Ethiopia should use these manuals in health care facilities in primary health care, community, and other settings. Furthermore, information on cognition and functional status is essential, and an appropriate diagnostic framework for depression in cognitively impaired older individuals is required.

## Strength & limitation

The included studies utilized a consistent set of diagnostic instruments to measure outcome variables, and this helped them to produce unbiased estimates of the overall prevalence of depression among older adults in Ethiopia. The overall prevalence of depression among older adults was derived only from studies conducted in four regional states (counties) of Ethiopia. Therefore, the findings of the present study should be interpreted with caution because the included studies only examined fewer than half of all the regional states of Ethiopia.

## Electronic supplementary material

Below is the link to the electronic supplementary material.

Supplementary Material 1. PRISMA Checklist

Supplementary Material 2. Subgroup analysis (setting)

Supplementary Material 3. Egger’s test

Supplementary Material 4. (Female)

Supplementary Material 5. (No formal edu)

Supplementary Material 6. (Chronic ds)

Supplementary Material 7. (Social support)

## Data Availability

All data generated or analysed during this study are included in this manuscript.
