# Supplementary material for: Prevalence and factors associated with depression among older adults in the case of a low-income country, Ethiopia: a systematic review and meta-analysis
Source: BMC Psychiatry. 2022 Nov 1;22:675. doi: 10.1186/s12888-022-04282-7 (PMC9624003; doi:10.1186/s12888-022-04282-7)
Supplement: Supplementary file 3 — Supplementary Material 3. Egger’s test [file 12888_2022_4282_MOESM3_ESM.docx]

Additional file 3: Egger’s test to assess publication bias in the included studies, 2021

| Standard  effect | Coefficient | Standard  error | t | p>\|t\| | 95% Confidence Interval |
| --- | --- | --- | --- | --- | --- |
| Slop | 3.66 | 0.23 | 15.90 | 0.00 | 3.14, 4.18 |
| Bias | -0.22 | 2.37 | -0.09 | 0.93 | -5.58, 5.15 |
