# Supplementary material for: Prevalence and factors associated with depression among older adults in the case of a low-income country, Ethiopia: a systematic review and meta-analysis
Source: BMC Psychiatry. 2022 Nov 1;22:675. doi: 10.1186/s12888-022-04282-7 (PMC9624003; doi:10.1186/s12888-022-04282-7)
Supplement: Supplementary file 5 — Supplementary Material 5. (No formal edu) [file 12888_2022_4282_MOESM5_ESM.docx]

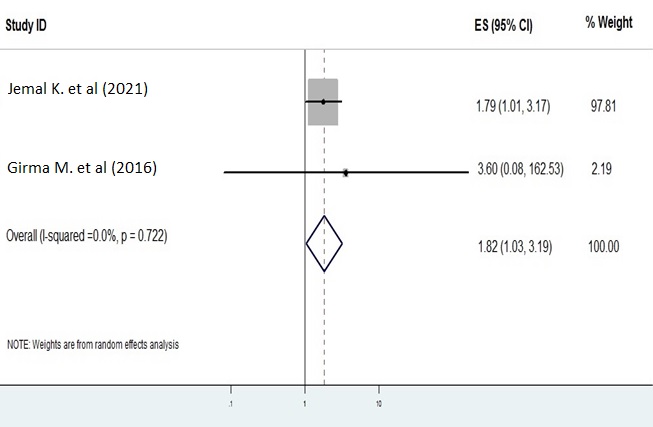
 Additional file 5: Forest plot of the adjusted odds ratios with corresponding 95% CIs of studies on the association of no formal education with depression among older adults in Ethiopia, 2021.
