# Supplementary material for: Prevalence and factors associated with depression among older adults in the case of a low-income country, Ethiopia: a systematic review and meta-analysis
Source: BMC Psychiatry. 2022 Nov 1;22:675. doi: 10.1186/s12888-022-04282-7 (PMC9624003; doi:10.1186/s12888-022-04282-7)
Supplement: Supplementary file 7 — Supplementary Material 7. (Social support) [file 12888_2022_4282_MOESM7_ESM.docx]

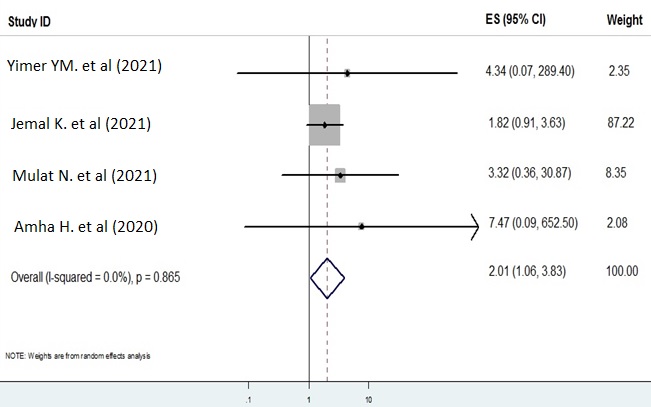


Additional file 7: Forest plot of the adjusted odds ratios with corresponding 95% CIs of studies on the association of social support with depression among older adults in Ethiopia, 2021.
